# Supplementary material for: Flexible neural connectivity under constraints on total connection strength
Source: PLoS Comput Biol. 2020 Aug 3;16(8):e1008080. doi: 10.1371/journal.pcbi.1008080 (PMC7425997; doi:10.1371/journal.pcbi.1008080)
Supplement: S1 Figs — (PDF) [file pcbi.1008080.s001.pdf]

# Supporting Information: Flexible neural connectivity under constraints on total connection strength

Gabriel Koch Ocker[1*], Michael A. Buice[1, 2],

**1** Allen Institute for Brain Science, Seattle, WA, USA
**2** Department of Applied Mathematics, University of Washington, Seattle, WA, USA

*gkocker@bu.edu

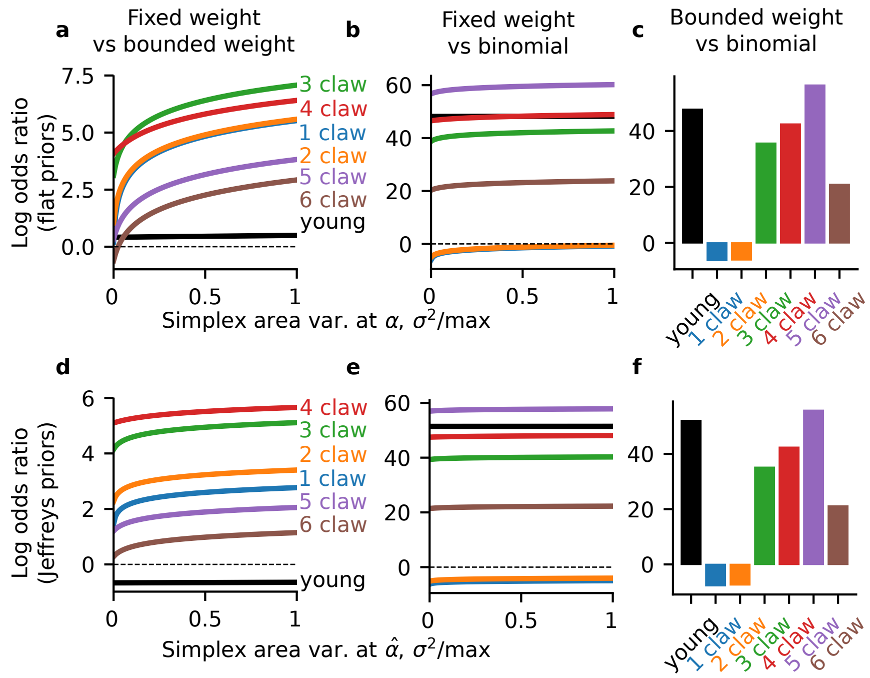


**S1 Fig A. Log odds ratios for larval Kenyon cell output degrees.** (**a**) Log posterior odds ratio for the simplex area distribution vs the simplex volume distribution, under a flat prior for the parameter $\alpha$. All posterior odds are computed by the Laplace approximation—a gaussian approximation for the likelihood in $\alpha$. The Laplace approximation yields point estimates of the posterior odds for the simplex volume and zero-truncated binomial models (Model comparison: bounded net weight model, Model comparison: zero-truncated binomial model). For the simplex area model, this yields lower and upper bounds for the posterior odds (Model comparison: fixed net weight model). Those bounds are parameterized by the variance of that gaussian, $\sigma^2_\alpha$. For each cell type, we varied $\sigma^2_\alpha$ between its lower and upper bounds and computed the posterior odds at each $\sigma^2_\alpha$. Since those bounds differ between cell types, we normalized them to compare the odds ratios. (**b**) Same as a), for the simplex area vs the zero-truncated binomial model. (**c**) Posterior odds ratio for the simplex volume vs the zero-truncated binomial model. (**d-f**) Same as (a-c), but with the Poisson Jeffreys prior for $\alpha$, $p(\alpha) \propto 1/\sqrt{\sigma}$.

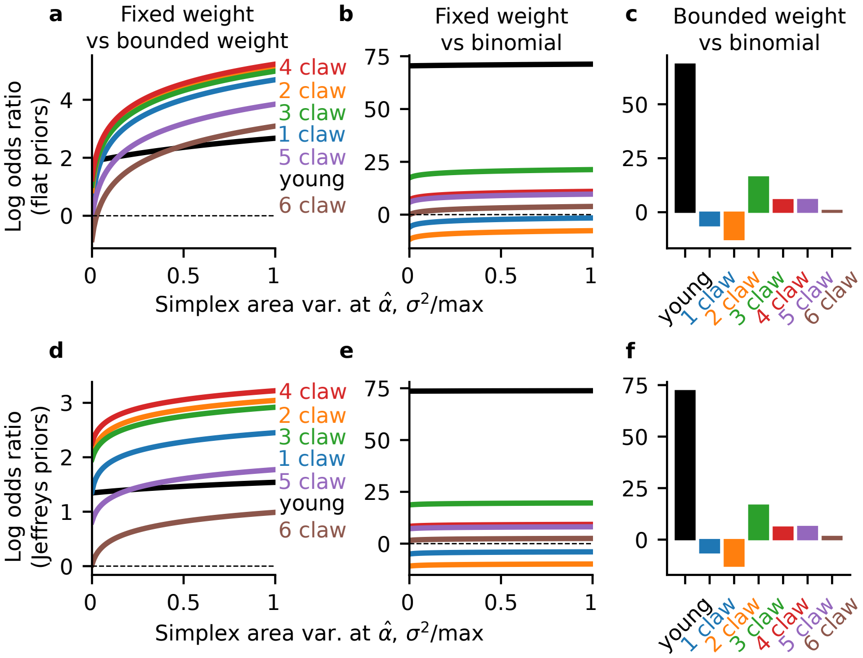


**S1 Fig B. Log odds ratios for larval Kenyon cell input degrees.** Same as S1 Fig A, but for larval Kenyon cell inputs.

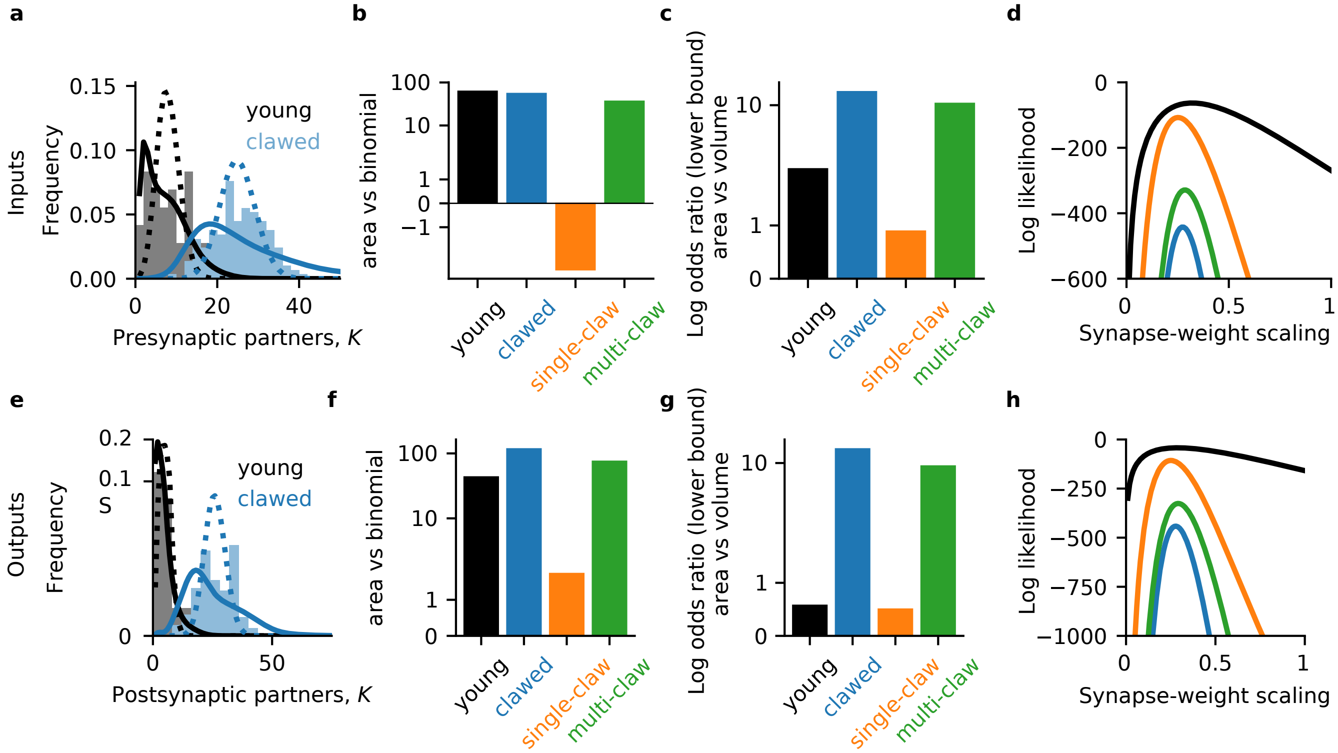


**S1 Fig C. Larval Kenyon cell axo-axonal connectivity.** (**a**) Input degree distributions for larval KCs. (**b**) Log odds ratio for the fixed net weight model vs the binomial wiring model. (**c**) Log odds ratio for the fixed net weight model vs bounded net weight model. (**d**) Likelihood as a function of the synapse-weight scaling parameter $\alpha$. **e-h**) Same as (**a-d**) for KC axo-axonal outputs.

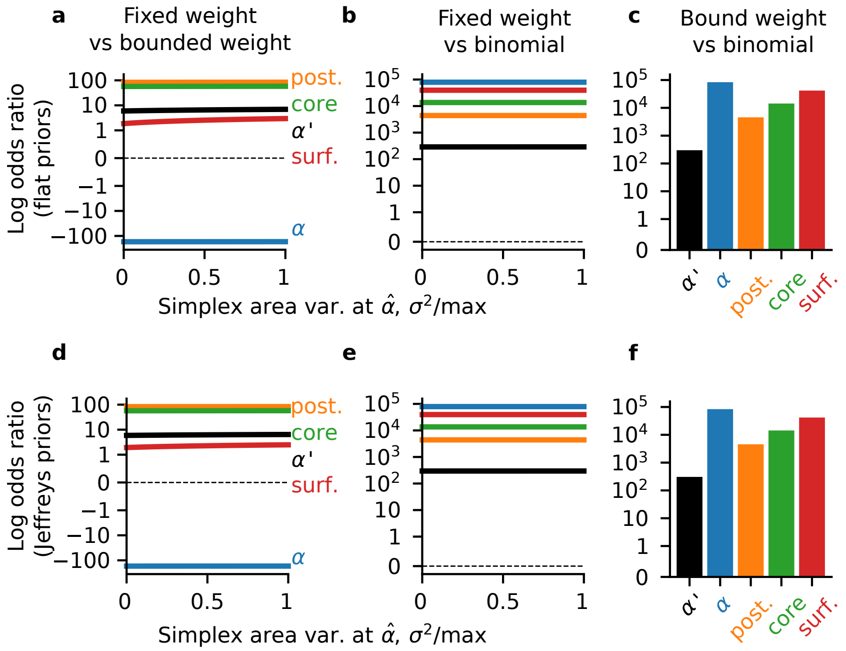


**S1 Fig D. Log odds ratios for adult Kenyon cell output degrees.** Same as S1 Fig A, but for adult Kenyon cell outputs.

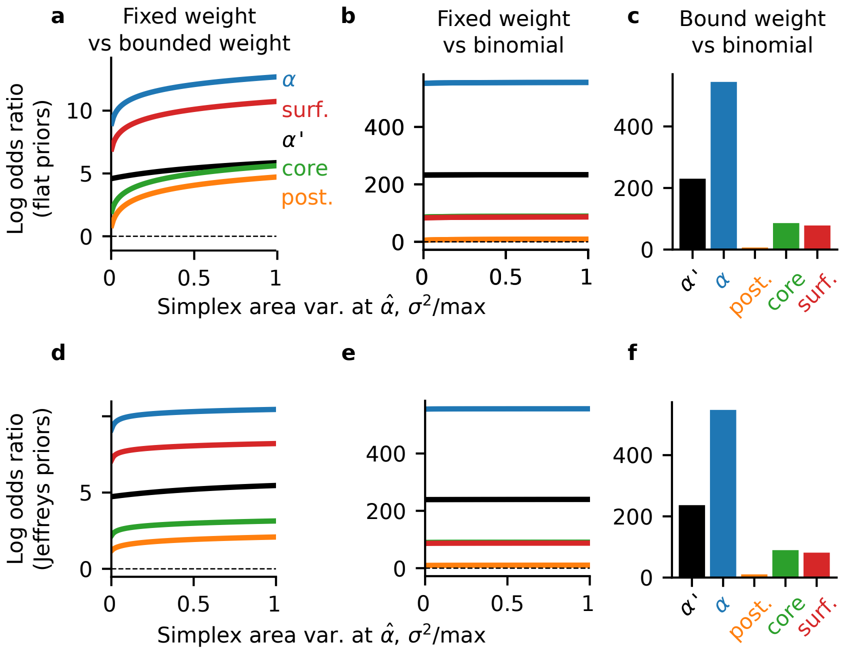


**S1 Fig E. Log odds ratios for adult Kenyon cell input degrees.** Same as S1 Fig B, but for adult Kenyon cells.

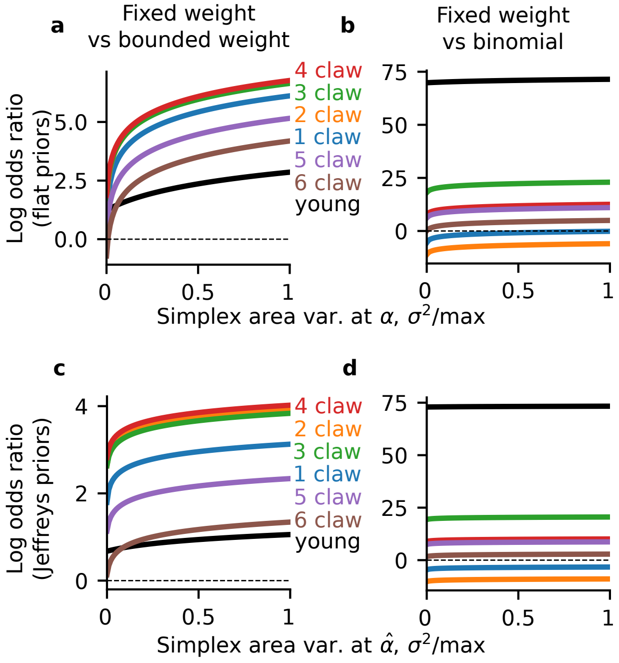


**S1 Fig F. Log odds ratios for larva Kenyon cell input degrees with 1-norm surface area model.** Same as S1 Fig B, but for the 1-norm simplex surface area model (Distances in synaptic configuration space).

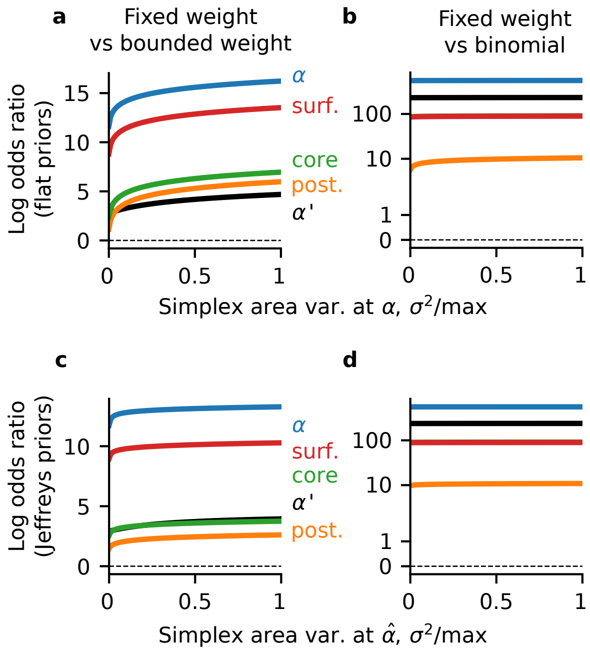


**S1 Fig G. Log odds ratios for adult Kenyon cell input degrees with 1-norm surface area model.** Same as S1 Fig E, but for the 1-norm simplex surface area model (Distances in synaptic configuration space).

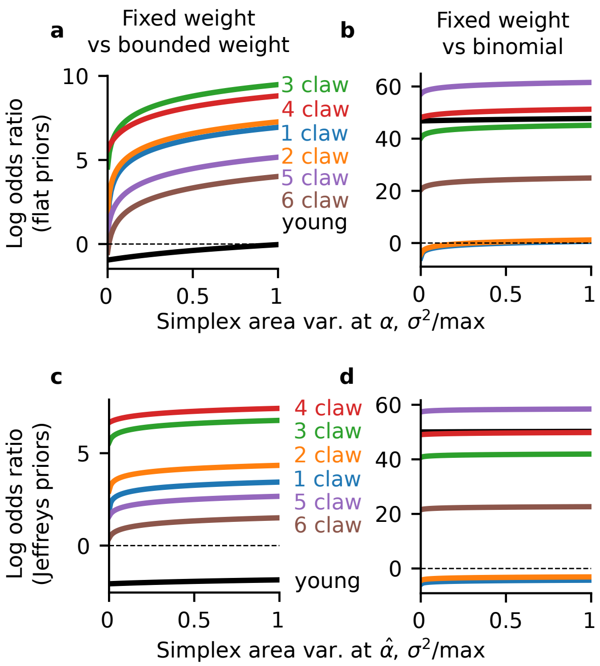


**S1 Fig H. Log odds ratios for larva Kenyon cell output degrees with 1-norm surface area model.** Same as S1 Fig A, but for the 1-norm simplex surface area model (Distances in synaptic configuration space).

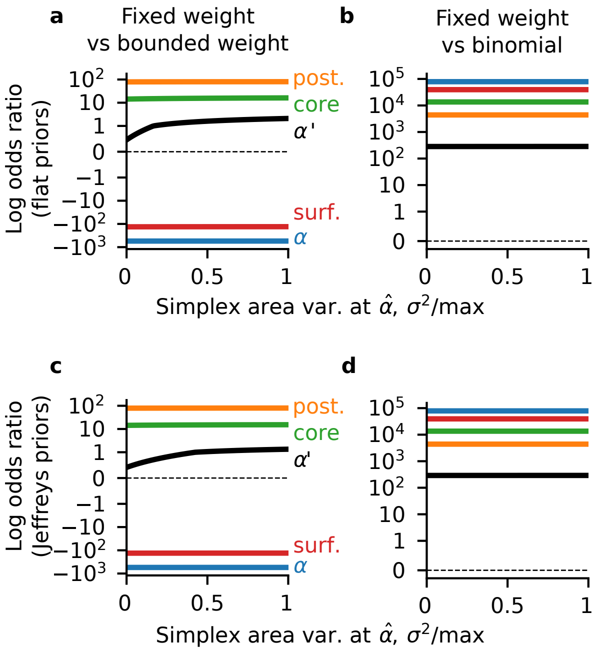


**S1 Fig I. Log odds ratios for adult Kenyon cell output degrees with 1-norm surface area model.** Same as S1 Fig D, but for the 1-norm simplex surface area model (Distances in synaptic configuration space).

## Materials and methods

Here we reproduce the Methods sections referenced in the supporting figure captions; the content is the same as in the corresponding main text.

### Model comparison

Under equal prior likelihoods for two models $X$ and $Y$, the posterior likelihood ratio between two models, $X$ and $Y$ is

$$\frac{p_X(K|\bar{S})}{p_Y(K|\bar{S})} = \frac{\int d\alpha \; \prod_i p_X(K_i|\bar{S}_i, \alpha)p(\alpha)}{\int d\alpha \; \prod_i p_Y(K_i|\bar{S}_i, \alpha)p(\alpha)} \tag{1}$$

where $i$ indexes data points. We consider the Laplace approximations for the posterior odds, obtained by writing $p_X = \exp \ln p_X$ and Taylor expanding the log likelihood $\ln p_X$ in $\alpha$ around its maximum likelihood value,

$$\hat{\alpha} = \arg\max_{\alpha} \prod_i p_X(K_i|\bar{S}_i, \alpha) \tag{2}$$

Truncating at second order then yields a tractable Gaussian integral over the unknown parameter:

$$\begin{aligned} \int d\alpha \; p(\alpha) \prod_i \; p_X(K_i|\bar{S}_i, \alpha) &= \int d\alpha \; p(\alpha) \prod_i \; \exp \ln p_X(K_i|\bar{S}_i, \alpha) \\ &\approx \int d\alpha \; p(\alpha) \prod_i \; p_X(K_i|\bar{S}_i, \hat{\alpha}) \exp \frac{-(\alpha - \hat{\alpha})^2}{2\sigma_i^2} \end{aligned} \tag{3}$$

where the integrals run over the allowed range for $\alpha$ and

$$\sigma_i^2 = -\left.\frac{\partial^2}{\partial \alpha^2} \ln p_X(K_i|\bar{S}_i, \alpha)\right|_{\hat{\alpha}} \tag{4}$$

Under a flat prior for non-negative $\alpha$, the marginal likelihood is:

$$p_X(K|\bar{S}) \approx \left( \prod_i p_X(K_i|\bar{S}_i, \hat{\alpha}) \right) \sqrt{\frac{\pi\sigma^2}{2}} \left( 1 + \mathrm{Erf}\left( \frac{\hat{\alpha}}{\sqrt{2\sigma^2}} \right) \right) \tag{5}$$

where $1/\sigma^2 = \sum_i 1/\sigma_i^2$. The simplex volume distribution is a truncated Poisson; we might reasonably use the Jeffreys prior for the Poisson distribution, $p(\alpha) \propto 1/\sqrt{\alpha}$. In that case, the marginal likelihood is

$$p_X(K|\bar{S}) \approx \left( \prod_i p_X(K_i|\bar{S}_i, \hat{\alpha}) \right) \frac{\pi\sqrt{\hat{\alpha}}}{2} \exp\left( -\frac{\hat{\alpha}^2}{4\sigma^2} \right) \left( \mathcal{I}_{-\frac{1}{4}}\left( \frac{\hat{\alpha}^2}{4\sigma^2} \right) + \mathcal{I}_{\frac{1}{4}}\left( \frac{\hat{\alpha}^2}{4\sigma^2} \right) \right) \tag{6}$$

where $\mathcal{I}_a$ is the modified Bessel function of the first kind. We will drop the indices on $K, \bar{S}$ in most of the remaining sections, reintroducing them where necessary.

### Model comparison: bounded net weight model

Under a bounded net weight, the degree distribution is:

$$p_V(K|\bar{S}, \alpha) = \frac{(\alpha\bar{S})^K}{Z_V(\bar{S}, \alpha)K!} \tag{7}$$

The normalization constant $Z$ is

$$Z_V(\bar{S}, \alpha) = \sum_{K=1}^{\infty} \frac{(\alpha \bar{S})^K}{K!} = \exp\left(\alpha \bar{S}\right) - 1 \tag{8}$$

so the simplex volume distribution is a zero-truncated Poisson distribution. We will make a Laplace approximation for the simplex volume distribution around $\hat{\alpha}$, leading to the posterior odds Eq 5 (for a flat prior on non-negative $\alpha$) or Eq 6 (for the Poisson Jeffreys prior). To calculate the Laplace approximation for the posterior odds we need $\hat{\alpha}$ and $\sigma^2$. The derivatives of $\ln p_V$ can be calculated directly (again dropping indices over measurements),

$$\begin{aligned} \frac{\partial}{\partial \alpha} \ln p_V(K|\bar{S}, \alpha) &= \frac{K}{\alpha} + \bar{S}\left(\frac{1}{1 - \exp \alpha \bar{S}} - 1\right) \\ \frac{\partial^2}{\partial \alpha^2} \ln p_V(K|\bar{S}, \alpha) &= -\frac{K}{\alpha^2} + \left(\frac{\bar{S}}{2} \operatorname{Csch}\left(\frac{\alpha \bar{S}}{2}\right)\right)^2 \end{aligned} \tag{9}$$

So we have

$$\sigma^2 = \left(\sum_i \left(\frac{K_i}{\hat{\alpha}^2} - \left(\frac{\bar{S}_i}{2} \operatorname{Csch}\left(\frac{\hat{\alpha}\bar{S}_i}{2}\right)\right)^2\right)^{-1}\right)^{-1} \tag{10}$$

and the maximum likelihood solution for $\alpha$ satisfies

$$0 = \sum_i \frac{K_i}{\hat{\alpha}} - \frac{\bar{S}_i}{1 - \exp \hat{\alpha} \bar{S}_i} \tag{11}$$

## Model comparison: fixed net weight model

Under the fixed net synaptic weight, our model is that the degree distribution is proportional to the surface area of the simplex:

$$p_A(K|\bar{S}, \alpha) = \frac{1}{Z_A(\bar{S}, \alpha)} \frac{(\alpha \bar{S})^{K-1}\sqrt{K}}{(K-1)!} \tag{12}$$

where

$$Z_A(\bar{S}, \alpha) = \sum_{K=1}^{\infty} \frac{(\alpha \bar{S})^{K-1}\sqrt{K}}{(K-1)!} \tag{13}$$

To calculate $\hat{\alpha}$ and $\sigma^2$ we need the derivatives of $\ln p_A$.

$$\frac{\partial}{\partial \alpha} \ln p_A = \frac{\partial}{\partial \alpha} \ln A - \frac{\partial}{\partial \alpha} \ln Z \tag{14}$$

where

$$\frac{\partial}{\partial \alpha} \ln A = \frac{K-1}{\alpha} \tag{15}$$

and we use the identity

$$\frac{\partial}{\partial \alpha} \ln Z = \frac{\frac{\partial}{\partial \alpha} Z}{Z} \tag{16}$$

We next bound $\frac{\partial}{\partial \alpha} Z$.

$$\begin{aligned} \frac{\partial}{\partial \alpha} Z &= \sum_{K=2}^{\infty} \frac{(\alpha \bar{S})^{K-2}}{(K-2)!} \sqrt{K} S \\ &= S \sum_{K=1}^{\infty} \frac{(\alpha \bar{S})^{K-1}}{(K-1)!} \sqrt{K} \sqrt{1 + \frac{1}{K}} \end{aligned} \tag{17}$$

For $K \geq 1$, $\sqrt{1+1/K}$ is bounded above by $\sqrt{2}$ and below by 1. So,

$$SZ < \frac{\partial}{\partial\alpha} Z < \sqrt{2} SZ \tag{18}$$

Inserting these into the critical point equation for $\hat{\alpha}$ provides the bounds:

$$\frac{\sum_i (K_i - 1)}{\sqrt{2}\sum_i \bar{S}_i} < \hat{\alpha} < \frac{\sum_i (K_i - 1)}{\sum_i \bar{S}_i} \tag{19}$$

We will also need the curvature of $\ln p_A$ w.r.t. $\alpha$ at $\hat{\alpha}$:

$$\sigma^2 = -\frac{\partial^2}{\partial\alpha^2} \ln p_A \tag{20}$$

Similarly to the first derivative,

$$\frac{\partial^2}{\partial\alpha^2} \ln p_A = \frac{\partial^2}{\partial\alpha^2} \ln A - \frac{\partial^2}{\partial\alpha^2} \ln Z \tag{21}$$

where

$$\frac{\partial^2}{\partial\alpha^2} \ln A(K|\bar{S}, \alpha) = -\frac{(K-1)}{\alpha^2} \tag{22}$$

We use the identity

$$\frac{\partial^2}{\partial\alpha^2} \ln Z = \frac{\frac{\partial^2}{\partial\alpha^2} Z}{Z} - \frac{\left(\frac{\partial}{\partial\alpha} Z\right)^2}{Z^2} \tag{23}$$

The curvature of $Z$ is

$$\begin{aligned} \frac{\partial^2}{\partial\alpha^2} Z &= S^2 \sum_{K=3}^{\infty} \frac{(\alpha\bar{S})^{K-3}}{(K-3)!} \sqrt{K} \\ &= S^2 \sum_{K=1}^{\infty} \frac{(\alpha\bar{S})^{K-1}}{(K-1)!} \sqrt{K} \sqrt{1 + \frac{2}{K}} \end{aligned} \tag{24}$$

The final term $\sqrt{1+\frac{2}{K}}$ is bounded above by $\sqrt{3}$ and below by 1, so

$$S^2 Z < \frac{\partial^2}{\partial\alpha^2} Z < \sqrt{3} S^2 Z \tag{25}$$

Defining upper and lower bounds for $\frac{\partial^2}{\partial\alpha^2} \ln Z$ using the upper and lower bounds of the first and second terms in Eq 23 yields:

$$-S^2 < \frac{\partial^2}{\partial\alpha^2} \ln Z < (\sqrt{3} - 1) S^2 \tag{26}$$

The upper bound for $\frac{\partial^2}{\partial\alpha^2} \ln Z$ provides an upper bound for $\sigma^2$, while neglecting $Z$ provides a lower bound for $\sigma^2$ (since $Z \geq 1$ from Eq 13, so that $\ln Z \geq 0$):

$$\frac{K-1}{\alpha^2} \leq \sigma^2 < \frac{K-1}{\alpha^2} + (\sqrt{3} - 1) S^2 \tag{27}$$

The posterior odds for the simplex area are:

$$\int d\alpha\, p(\alpha) \prod_i p_A(K_i|\bar{S}_i, \alpha) \approx \left( \prod_i p_A(K_i|\bar{S}_i, \hat{\alpha}) \right) \int d\alpha\, p(\alpha) \exp\left( \frac{(\alpha - \hat{\alpha})^2}{2\sigma^2} \right) \tag{28}$$

where $\sigma^2 = 1/\sum_i 1/\sigma_i^2$. We use the upper and lower bounds for $\sigma_i^2$ to define upper and lower bounds, respectively, for the likelihood's variance:

$$\begin{aligned} \sigma_U^2 &= \left( \sum_i \left( \frac{(K_i - 1)}{\hat{\alpha}^2} + \left(\sqrt{3} - 1\right) \bar{S}_i^2 \right)^{-1} \right)^{-1} \\ \sigma_L^2 &= \left( \sum_i \left( \frac{(K_i - 1)}{\hat{\alpha}^2} \right)^{-1} \right)^{-1} \end{aligned} \quad (29)$$

We compute $\hat{\alpha}$ numerically by maximizing the likelihood, and compute $p_A(K_i|\bar{S}_i, \hat{\alpha})$ also numerically, estimating $Z$ by ranging over $K = 1$ to $2 \max_i \bar{S}_i$.

## Bounds for the posterior odds of the fixed net weight model

The derivative of the posterior odds under the flat prior, Eq 5, with respect to $\sigma$ is proportional to

$$1 - \sqrt{\frac{2}{\pi}} \frac{\alpha}{\sigma} \exp\left(-\frac{\alpha^2}{2\sigma^2}\right) + \mathrm{Erf}\left(\frac{\alpha}{\sqrt{2}\sigma}\right) \quad (30)$$

Since $\alpha > 0$ and $\sigma > 0$, the last term is bounded between 0 and 1. The middle term is proportional to the form $x \exp(-x^2/2)$, which is maximized by $1/\sqrt{e}$ at $x = 1$. Since $\sqrt{2/\pi e} < 1$, the middle term is less than 1 and the derivative of the posterior odds under a flat prior for $\alpha$, with respect to $\sigma$, is non-negative. The upper bound for $\sigma^2$ thus provides an upper bound on the posterior odds. We see that the posterior likelihood $\prod_i p_A(K_i|\bar{S}_i)$ increases from $\sigma_L^2$ to $\sigma_U^2$ (reflected in the log posterior odds ratio for the simplex volume vs the simplex area, S1 Figs 1, 2, 4-9).

The derivative of the posterior odds under the Poisson Jeffreys prior, Eq 6, with respect to $\sigma^2$, is proportional to

$$\begin{aligned} -\frac{\alpha^2}{2\sigma^3} \exp\left(-\frac{\alpha^2}{4\sigma^2}\right) \Bigg( \mathcal{I}_{-\frac{5}{4}}\left(\frac{\alpha^2}{4\sigma^2}\right) + \mathcal{I}_{-\frac{3}{4}}\left(\frac{\alpha^2}{4\sigma^2}\right) + 2\mathcal{I}_{-\frac{1}{4}}\left(\frac{\alpha^2}{4\sigma^2}\right) \\ + 2\mathcal{I}_{\frac{1}{4}}\left(\frac{\alpha^2}{4\sigma^2}\right) + \mathcal{I}_{\frac{3}{4}}\left(\frac{\alpha^2}{4\sigma^2}\right) + \mathcal{I}_{\frac{5}{4}}\left(\frac{\alpha^2}{4\sigma^2}\right) \Bigg) \end{aligned} \quad (31)$$

We saw that the posterior odds for the simplex area distribution also increased with $\sigma^2$ for the Poisson Jeffreys prior.

## Model comparison: zero-truncated binomial model

The marginal likelihood for the zero-truncated binomial with distribution $p_B$ is

$$\int dq\, p(q) \prod_i p_B(K_i|N, q) \approx \left( \prod_i p_B(K_i|N, \hat{q}) \right) \int dq\, p(q) \prod_i \exp\left( -\frac{(q - \hat{q})^2}{2\sigma^2} \right) \quad (32)$$

where $p_B(K_i|N, q) = \binom{N}{K_i} \frac{q^{K_i}(1-q)^{N-K_i}}{1-(1-q)^N}$. For connections to larval KCs, we used the total number of traced projection neurons (PNs) and KCs as the binomial parameter $N$, averaged over the two sides of the brain [1]. For projections from larval KCs, we used the total number of KCs and output neurons, averaged over the two sides, as $N$. For projection to adult KCs, we used the number of Kenyon cells plus 150 (the estimated number of olfactory PNs) as $N$ [2]. For projections from adult KCs, we used the number of KCs and output neurons labelled in the data as $N$.

The variance with respect to $q$ is determined as in Eq (4). The derivates of $\ln p_B$ are, again dropping indices on $K$,

$$\begin{aligned}\frac{\partial}{\partial q}\ln p_B &= \frac{K}{q} - \frac{N-K}{1-q} - \frac{N(1-q)^{N-1}}{1-(1-q)^N} \\ \frac{\partial^2}{\partial q^2}\ln p_B &= -\frac{K}{q^2} + \frac{N-K}{(1-q)^2} + \frac{N\left(N-1+(1-q)^N\right)(1-q)^{N-2}}{\left((1-q)^N-1\right)^2}\end{aligned} \tag{33}$$

The maximum likelihood parameter $\hat{q}$ for the zero-truncated binomial, with $M$ samples of $K$, each with $N$ trials, obeys:

$$\frac{\hat{q}}{1-(1-\hat{q})^N} = \frac{\sum_{i=1}^{M} K_i}{MN} \tag{34}$$

and the variance at $\hat{q}$ is

$$\sigma^2 = \left(\sum_i \left(\frac{K_i}{\hat{q}^2} - \frac{N-K_i}{(1-\hat{q})^2} - \frac{N\left(N-1+(1-\hat{q})^N\right)(1-\hat{q})^{N-2}}{\left((1-\hat{q})^N-1\right)^2}\right)^{-1}\right)^{-1} \tag{35}$$

## Distances in synaptic configuration space

Above we assumed that synaptic weight configurations could travel between different points in the synaptic weight space along straight lines, endowing the $K$-dimensional synaptic weight space with a Euclidean (or 2-) norm. This amounts to assuming that synaptic weights can vary together. This could be interpreted, for example, as allowing a unit of synaptic weight (a receptor, perhaps) to be transferred directly between connections. An alternative is to assume that synaptic weights must move separately, which corresponds endowing the synaptic weight space with the 1-norm. In the above interpretation this would mean separating the removal of a receptor from one synapse from the addition of a receptor to another synapse. This changes the surface area of the simplex, since its inner radius is $\bar{J}/K$ rather than $\bar{J}/\sqrt{K}$:

$$A_1 = \frac{(\bar{J}K^p)^{K-1}K}{(K-1)!} \tag{36}$$

Changing the norm for the synaptic weights leaves the above calculation of the posterior odds for the fixed net weight model mostly unchanged. The factors of $\sqrt{K}$ in the normalization constant are replaced by $K$; this removes the square roots in the derivation of the upper bound for the variance with respect to $\alpha$ so that

$$\frac{K-1}{\alpha^2} \leq \sigma^2 < \frac{K-1}{\alpha^2} + 2S^2 \tag{37}$$

The optimal number of connections can be calculated in the same manner as previously. The derivative of $A_1$ with respect to $K$ is (to order $1/K$):

$$\frac{\partial A_1}{\partial K} \approx \frac{(\bar{J}K^p)^{K-1}}{(K-1)!}\left(K\ln \bar{J}K^p + (K-1)p - K\left(\ln(K-1) + \frac{1}{2(K-1)} + \mathcal{O}\left(\frac{1}{K^2}\right)\right) + 1\right) \tag{38}$$

At a critical point in $K$, truncating $\mathcal{O}(1/K^2)$ and higher-order terms yields

$$\bar{J} \approx K^{-p}(K-1)\exp\left(\frac{1}{K(K-1)} - p\frac{K-1}{K}\right) \tag{39}$$

## References

1. Eichler K, Li F, Litwin-Kumar A, Park Y, Andrade I, Schneider-Mizell CM, et al. The complete connectome of a learning and memory centre in an insect brain. Nature. 2017;548(7666):175–182. doi:10.1038/nature23455.

2. Jefferis GS, Marin EC, Stocker RF, Luo L. Target neuron prespecification in the olfactory map of Drosophila. Nature. 2001;414(6860):204–208. doi:10.1038/35102574.
