# Supplementary material for: Flexible neural connectivity under constraints on total connection strength
Source: PLoS Comput Biol. 2020 Aug 3;16(8):e1008080. doi: 10.1371/journal.pcbi.1008080 (PMC7425997; doi:10.1371/journal.pcbi.1008080)
Supplement: Attachment — Submitted filename: review_response.pdf [file pcbi.1008080.s002.pdf]

We thank the reviewers for their considered reading of our work. We believe their feedback has greatly improved the manuscript. In response, we have made these major revisions:

1. We have significantly revised the Introduction to clarify our general idea and its application to the mushroom body, including a new introductory figure panel of the mushroom body circuit.
2. We have reorganized the Results to focus sequentially on the larval and then adult mushroom bodies.
3. We have added a new analysis of the inputs onto mushroom body output neurons of the larva, complementing the previous analysis of the earlier stage of that circuit (Kenyon cells).
4. We have gone through and revised the text, especially in the Results, to move mathematical jargon to the Methods section when possible while still communicating our main idea clearly, and to clarify the motivation behind model assumptions.

Below we address individual reviewer comments. We quote reviewers' comments in blue and revisions to the manuscript in red.

Reviewer 1

Major points

1: The biological basis of the model. It is difficult for the reader to relate the computational work with the biology when reading this manuscript. As it stands, the biology stands alone and it is never referred to by the authors when introducing difficult mathematical concepts. For example: Line 25-30: In simple neural network models, constraints define a space of possible synaptic weights (the "constraint space"), while the synaptic weights that perform a particular computation define another space (the "computational space"). The intersection of constraint and computational spaces defines the allowed synaptic weight (the "solution space"). While this (kind of) makes sense, it would be useful to remind the reader of the mushroom body analogy: what synapses and which computation are considered here? Another example is "flexibility". Are the authors referring to the flexibility of the projection neuron/Kenyon cell synapse or the Kenyon cell/output neuron synapse? Or some other flexibility? What do they mean? We need to know.

We have rewritten the Introduction to focus more on the mushroom body circuitry as suggested, and to clarify what we mean by flexibility:

> The connectivity of neural circuits, together with their intrinsic dynamics, determines their computation. A goal of systems neuroscience is to uncover and describe these computational mechanisms in specific circuits. For example, associative memory is a quintessential neural computation [1–3]. In cerebellar and cerebral cortices, random connectivity may form high-dimensional representations to facilitate associative memory [4–7], and the synaptic weight distributions of Purkinje cells and cortical pyramidal neurons are consistent with optimal associative memory in a perceptron model [8–12].
>
> At the same time, neuronal connectivity is constrained by physiological limits like resource limitations and homeostatic requirements. The total strength of synaptic connections between two neurons is limited by the amount of receptor and neurotransmitter available and the size of the synapse [13]. Homeostatic synaptic scaling in pyramidal neurons of mammalian cortex and hippocampus regulates their total excitatory [14–17] and inhibitory [18–22] synaptic input strengths to regulate activity levels [23].

The neural connectivity necessary for a given computation must exist or develop within these physiological constraints. Furthermore, the computations performed by a circuit may require modification based on exposure to the environment and the needs of the organism. Physiological constraints could conflict with a required circuit configuration and pose a challenge to computational learning. It might thus be advantageous for circuits to enable a broad array of computations, given a fixed constraint. We refer to connectivity patterns that can enable many computations as "flexible" under a constraint. Here we seek to understand whether this flexibility can predict neural connectivity patterns.

We examine the interaction between constraints and computations in the mushroom body, a cerebellum-like associative memory center in *Drosophila melanogaster* and other insects [24, 25]. Mushroom bodies are composed largely of Kenyon cells (KCs), which receive input from sensory projection neurons (PNs; Fig. 1a) . KCs also connect recurrently to each other, receive inhibitory and modulatory inputs, and project to mushroom body output neurons (MBONs). A combinatorial code for odorants in KCs [26–28] forms a substrate for associative learning at KC-MBON synapses [24, 29]. In *D. melanogaster*, neurons from a variety of circuits exhibit homeostatic regulation of connectivity during growth from the first instar larva to the third instar larva. This includes a homeostatic regulation of mechanosensory receptive fields [30], functional motor neuron outputs [31], and nociceptive projections [32]. Changes in inputs to central motor neurons elicit structural modifications to their dendrites that homeostatically maintain input levels [33]. Finally, changes in olfactory PN activity lead to homeostatic compensations in the number and size of their output synapses in the mushroom body [34–36]. Connections in the mushroom body also are generally limited to a few synapses per connection [37, 38]. We thus hypothesize that mushroom body connectivity in *D. melanogaster* might be structured to be computationally flexible under physiological constraints.

Consider the connections from one KC to its K targets. We will describe each projection by one synaptic weight, summed across synapses if the projection is multi-synaptic, so that this KC's output weight configuration is a point in a K-dimensional synaptic weight space. Regions of this space might correspond to weight configurations that support different learned associations or computations (Fig. 1b; "computation spaces"). A physiological or homeostatic constraint, such as those discussed above, also defines a region of allowed connectivity configurations (Fig. 1b; "constraint space"). If a constraint is very tight, it might only allow a few configurations, and even disallow computationally useful configurations as in Fig. 1b, where the constraint and computation spaces do not overlap. In order for the KC to be both healthy and computationally useful, its connectivity must lie in the intersection of the constraint space and a computation space. This is easier if the constraint space is large—if the KC's connectivity is flexible under its constraints. (Fig. 1c). "Flexibility" thus refers to the number of possible joint configurations of all this KC's outputs and is a property of the neuron's full K-dimensional output connectivity rather than of an individual synapse. While we discussed the flexibility of a KC's output connectivity here, the same idea can be applied to its inputs, or those of MBONs, or of other neurons in other systems.

2: The mathematical jargon needs to be introduced and difficult concepts need to be digested. This manuscript is difficult to read for non-mathematicians. The authors are often vague and use a lot of technical terms without clearly defining them. This might not bother the specialized audience of PLoS Computational Biology, but the paper would be more appealing and would reach a broader audience if it was better written. I am listing a few examples of concepts that are unbeknownst to me and some sentences that are especially cryptic:

- Line 199: We computed the Laplace approximation to these model evidences.
- Line 228: The Jeffreys prior is invariant under monotone changes of variables, resolving this paradox.
- Moreover, it is especially important for the authors to explain their findings better rather than just referring to a figure and leaving the reader to their own device. For example:

- Line 209: In the larva, the simplex area model outperformed the binomial model for both young and clawed KCs (Figure 3C). What do the authors mean by "outperformed"? How can I reach this conclusion by looking at Figure 3C (a bar graph)?

There are many more such examples. I would recommend that the authors recruit a biologist on their team to try and make this manuscript readable for both mathematicians and biologists.

We have endeavored to remove mathematical jargon wherever possible throughout the manuscript, or to replace it with references to the Methods sections, including each of those examples. In addition, we have added a definition of the log odds that we use to compare models:

> To quantitatively compare how two models explained the data, we computed their log evidence ratio (log odds). The log odds for the fixed net weight model p_A versus the bounded net weight model p_V are
>
> $$L = \ln \frac{p_A(K|\bar{S})}{p_V(K|\bar{S})} \quad (1)$$
>
> and positive L favors the fixed net weight model, while negative L favors the bounded net weight model. For example, $L = 1$ means that the data are $\exp(1) \approx 2.72$ times more likely under the fixed net weight model than the bounded net weight model and $L = 10$ means the data are $\exp(10) \approx 22026.47$ times more likely under the fixed net weight model.

and clarified statements throughout the Results about the log odds favoring one model or another (previously using the phrase "outperformed").

3: Consistency in the biological data. While the biology used in this manuscript is sound, it is not clear to me why the authors using different systems, namely the larval and the adult mushroom body. Sometimes, the biology used to support their assumptions is yet borrowed from another system, hippocampal neurons for example. This lack of consistency makes the paper extremely confusing. Why not exploit one system, the larval mushroom body seems more appropriate here, and explain that system well and explicitly make the links between the biological system and the mathematical model.

We view the inclusion of data from multiple circuits—the larval and adult mushroom bodies—as a strength of this study; it demonstrates the generality of our approach. However, the reviewer's suggestion is a good one. We have reorganized the Results text and figures to focus on one circuit at a time. Figures 3 and 4 of the revised manuscript focus on the larval mushroom body, including a new analysis of inputs to the mushroom body output neurons. Figure 5 shows results for the adult mushroom body. In addition, we have added citations in the introduction demonstrating homeostatic regulation of projection neuron to Kenyon cell wiring (see the quoted Introduction revision under Point 1). To our knowledge, resource limitations and homeostatic regulation of connectivity have not been experimentally examined in other mushroom body synapses.

Reviewer 2

Major comments

1) The authors initially make a clear case for the intersection between the constraint space and the solution space, and posit that he number of configurations of synaptic unites (vesicles or postsynaptic receptors) corresponding to the number of synapses is a reasonable marker of computational flexibility under given constraints. They even provide toy examples of this notion (lines 64 to 72.) The reader then expects to see whether the number of synapses in biology is tuned to the utilize the calculated maximum number of configurations under the postulated constraints (bounded total weight vs. fixed total weight.) However, that is not what the authors show. What they show is whether the in- (and out-) degree distribution of KC?s follow the distribution under the constraints, without any consideration of the maximum number of configurations. In other words, the authors do a great job delineating the constraint space, but not for its intersection with solution space. Only at the end of the results section, they look

into the mode of the degree distributions (the supposed maximum number of configurations given the in/out degree and constraint). There, they show that the mode has a linear relationship with the number of synapses (K) in both models, with only the intercept (-1/2 vs. 2 in equation 12 and 13, respectively) being different between the two models. If this is the case, they should have examined the intercept in their experimental data, e.g. with a linear regression model.

Our original presentation of the toy examples of Fig. 1 indeed focused on the change in the maximally flexible number of configurations as the number of available synaptic weight units, N, changed. We thank the reviewer for pointing out that this led a reader to expect the subsequent analysis to focus on this. Our goal, however, was to focus on a more general observation: that the whole profile of the number of available configurations, as a function of the number of connections K, changes with N. This observation, together with the hypothesis that neurons might face a pressure towards flexible connectivity under constraints, motivated the probabilistic models for $p(K)$ that we tested. We have edited the abstract and the presentation of that section to reflect this focus:

> To measure how flexible the neuron is with M synaptic weight units and K partners, we can count possible connectivity configurations. Since the constraint treats all synaptic partners symmetrically, the number of possible configurations is given by the binomial coefficient "M choose K". For $M = 4$ and degree two, there are six possible configurations. With $M = 4$ and degree three, there are four possible configurations. Thus with the constraint of $M = 4$, the neuron is more flexible with two connections than three since there are more ways to satisfy the constraint. The neuron's flexibility under this constraint (of fixed total synaptic resources $M\Delta J$) is a function only of the number of synaptic partners K only. For different numbers of synaptic weight units M, the flexibility exhibits different profiles as a function of the degree K (Fig. 1f).

and expanded the introduction of the probabilistic models:

> Under our hypothesis of flexible computation under a constraint, there should be a pressure towards circuit structures with a large number of allowed synaptic weight configurations. There may be other competing pressures on the circuit architecture, which prevent it from being optimally flexible (achieving the largest number of possible configurations). To model the pressure towards flexibility, we thus stipulate that the probability of having K synaptic partners given resource limits $\bar{J}$ is proportional to the number of possible configurations, i.e. the volume of the weight space with K partners.

The more restricted observation, that the location of the optimally flexible degree also depends on the constraint (the number of units of synaptic weight available in Fig. 1, or the total or maximal total connection strength in Fig. 2), is then examined at the end of the Results. The reviewer suggests that we fit a line to data points in the $\bar{S}, K$ plane in order to test our theory. Choosing a cost function to optimize for the line fit implies a noise model for fluctuations around the line. Ordinary least squares regression, for example, corresponds to assuming a Gaussian noise model [39]. The model comparison we performed in Figs. 3-5 tests how well the models of Eqs. 5 and 8 describe both the location of the mode of the distribution and the fluctuations around it. We now state this in the Results:

Eqs. 14 and 15 reveal that to leading order, the model comparisons of Figs. 3-5 encapsulate linear fits to $\bar{S}$ as a function of K while accounting for the variability around that line predicted by each constraint.

2) It is not clear why the parameter α is introduced to relate synaptic units to synaptic strengths. Based on the theory developed before the experimental tests, it is expected that they consider parameters such as the total number of synaptic vesicles for each neuron (or the total magnitude of postsynaptic densities) as a their measure of total synaptic units ($\bar{J}$) and test their models against this, (i.e. how these units are distributed among synapses? Are the number of synapses tuned in such a way that the number of possible configurations of these units are maximized? etc.) But that's not the analysis provided in the manuscript. Instead, the total number of synapses S is assumed to be proportional to J with an unknown constant of proportionality α. In simple terms, the data is too coarse to be able to test the model. Maybe the authors have a cogent explanation for how the data is related to the model, but it is not obvious to me. Importantly, they test the two models against a binomial distribution, but there

is no α in the binomial case. Is it a fair comparison? It is also somewhat surprising why the binomial model is so poor compared to Poisson model, because the latter is a continuous approximation of the former. Am I missing anything here? There is also no comparison between binomial and the two models in testing for optimally flexible connectivity (figure 5.)

More detailed measurements of presynaptic resources, like numbers of vesicles, are possible from the electron microscopy data that allowed the published circuit reconstructions we used [37, 38]. To our knowledge, individual receptors (the postsynaptic resources we postulate) are not visible in the same data. Crucially, both pre- and postsynaptic constraints might not operate directly on the number of vesicles or receptors.

The parameter α is thus introduced precisely because of the uncertainty the reviewer describes: we do not have a precise measurement of the units of synaptic weight that are constrained (of $\bar{J}$). Our idea does not, however, require specifying the biophysical units or the molecular mechanisms of the constraint. Rather, it only requires the reasonable assumption that physiological and anatomical measures of connection strength are correlated. This assumption has been tested and been born out in mammalian pyramidal neurons, as discussed in the Results when it is introduced:

The published EM wiring diagrams of *D. melanogaster* mushroom bodies measure synaptic strengths by the count of synapses [37, 38]. While we are not aware of joint measurements of synapse counts and physiological connection strength in Kenyon cells, the relationship of anatomical and physiological measures of connection strength has been studied extensively in mammalian pyramidal neurons. There, synapse size and synapse strength are highly correlated [40–44].

In future work, a more detailed model of the relationship between anatomical and physiological measures of connection strength, perhaps using cable theory to account for the anatomical location of synapses, could replace the simple model $\bar{J} = \alpha \bar{S}$. For specific biophysical constraints, rather than the general ones we considered, a detailed model also of the intracellular signaling pathways involved could also be incorporated. We have added this to the Discussion:

Anatomical measures of connection strength

To test the hypothesis that neurons in the mushroom body are subject to a pressure towards flexible connectivity under constraints, we required measurements of the total input or output connection strength of these neurons. For this purpose, we used electron microscopic reconstructions of mushroom body circuitry [37, 38]. These published data contain anatomical measurements of connectivity: the number of synapses between neurons. The general types of constraint we considered (bounded or homeostatically fixed total connection strengths) might not operate directly on synapse counts. To account for this uncertainty, we assumed that synapse counts were proportional to the constrained total connection strength (Eq. 10) [37]. Spatially detailed, biophysical neuron models could in principle be used to account for synapse locations and the passive and active membrane conductances transforming anatomical connectivity into physiological connection strengths in specific neurons. In hippocampal pyramidal cells, cerebellar Purkinje cells, and Drosophila visual neurons, dendritic structures can compensate for signal decay systems [45–48]. If this is also the case in mushroom body neurons, detailed spatial models to relate anatomical and physiological connection strengths might not provide additional insight. Alternatively, additional information about the processes governing homeostatic synaptic scaling or synaptic resource limits could motivate models of a different functional form than Eq. 10 [49, 50].

The binomial null model we consider indeed does not have the same unknown parameter α as the others; rather the connection probability p is unknown. This is part of the reason that we performed a Bayesian model comparison, marginalizing out the unknown parameters of both the binomial and constraint models to measure the performance of the form of each model, rather than the performance at particular parameter values.

The Poisson model obtained from the bounded net synaptic weight is indeed an approximation to a binomial model, where M units of synaptic weight are allocated with some common probability p (as discussed in Methods: Measuring synaptic weight configuration spaces). The binomial model that we used, however, was one where each of N potential synaptic partners made a connection with probability p. Our original manuscript used N when

discussing both of those binomial models; to help prevent this confusion our revised manuscript uses M for the number of available vesicles. In addition, we point out the distinction between those two models when we introduce the binomial wiring model:

> This binomial wiring model assumes that each possible synaptic partner to a Kenyon cell has a fixed probability of making a connection, and that whether or not different potential partners actually connect is independent. In this binomial wiring model, each neuron's degree is conditioned on two things: N (the number of potential synaptic partners) and q (the connection probability). We used anatomical measurements for N (Methods: Zero-truncated binomial model) and took q as an unknown parameter for this model. Note that this is a binomial model for connections, in contrast to the binomial model for resource allocation of Fig. 1d-f.

Finally, that binomial wiring model is consistent with a linear relation between the number of synapses and number of connected neurons if the number of synapses per connection is chosen independently from the same distribution. It does not, however, directly correspond to any optimization or flexibility. We have added a paragraph pointing this out in that Results section:

> The binomial model we examined above does not depend on or model synapse counts. If it were augmented with a wiring process where each connection independently sampled a number of synapses, its total synapse count and number of connections would also be linearly related. Each of these three models are thus consistent with the same qualitative result. The model comparison we performed above tests which best explains the data, accounting for the variability around the mode described by each model (Figs. 3-5).

3) The description of the experimental data is very confusing. The authors should appreciate that PLoS Computational Biology is not a neuroscience journal, and even in a neuroscience journal with a wide audience it is not the case that all readers are fully familiar with the insect olfactory system. A schematic figure of mushroom body, its parts and the location of KC neurons, and their input/output will be very helpful. From the results (lines 137-156) it is not clear whether the input from projection neurons is included in the analysis or not. The same notion as in line 149 is repeated in line 154 ("KCs can be morphologically classified by their age", and "The age of adult KCs can also be classified morphologically.") This section requires a clear rewrite to my opinion, with an explicit explanation of what is being measured, what is exactly accounting for the in-degree, what is out-degree, how many neurons are being studied, how much sub-sampling happens in the measurements? How sensitive the results are to sub-sampling? etc.

We thank the reviewer for pointing this out. We have significantly revised the introduction and results sections to focus more on the biological circuitry, including a new mushroom body circuit diagram (Fig. 1a). We also now describe our analysis of the larval and adult mushroom bodies in separate sections, introducing in each the morphological classification of larval or adult KCs. For the larval KCs, there is no subsampling; Eichler et al. traced all the inputs and outputs of each KC [37]. For the adult, the electron microscopy volume included only KC axons, so it does not include the PN inputs. We describe data inclusion at the beginning of the Results section for each circuit. For the larva:

> We first examined KCs outputs in the first instar larva, using the complete synaptic wiring diagram of its 223 KCs (110 on the left and 113 on the right) from Eichler, Li, Litwin-Kumar et al. [37]. We excluded projections to the inhibitory APL neuron and the modulatory dopaminergic, and octopaminergic neurons as well as interneurons so the out-degrees measure the number of postsynaptic KCs and MBONs of each KC. (There are 48 MBONs.) We obtained similar results as reported here, however, when including those other synapses. As in previous studies, we used only reliable multi-synapse connections (but obtained similar results as reported when including single-synapse connections) [37,38]. Larval KCs can be morphologically classified by their age. The dendrites of mature KCs form claws around projection neuron axons. The 78 young KCs do not have claws, and the 36 single-claw KCs are older than the 109

> multi-claw KCs [37]. We observed a wide range of numbers of presynaptic partners to KCs, with very different out-degree distributions for young and clawed KCs (Fig. 3a histograms).

And for the adult:

> To test the generality of our results, we turned to a related circuit: the adult *D. melanogaster* mushroom body. It contains the same types of cells as the larva, with the same overall types of connections (Fig. Fig1a), but in different numbers. Specifically, we examined a recent connectome of the alpha lobe of the adult mushroom body from Takemura et al. [38]. The alpha lobe is defined by KC axons, so the reconstruction does not include the PN inputs onto the alpha lobe KCs' dendrites. It contains the axons of 132 alpha prime lobe KCs and 949 alpha lobe KCs. Like in the larva, the age of adult KCs can be classified morphologically. KCs of the alpha prime lobe are born before KCs of the alpha lobe. In the alpha lobe, the 78 posterior KCs are born before the 480 surface KCs, which are in turn born before the 259 core KCs [51, 52]

Minor comments

1) It would be helpful if the authors explain how they obtain eq 7. For me it is clear, as the length of the norm vector of the simplex surface is J/sqrt(K) and hence moving in the direction of the norm vector by dJ/sqrt(K) is equivalent to changing J by dJ. But it helps with the clarity of the manuscript if it is explained it in the Methods.
We have added this calculation to the Methods section "Measuring synaptic weight configuration spaces"

2) The subtitle "Measuring spaces of allowed circuit configurations" in discussion is not clear at all. In fact the same topic in Methods is more clearly explained!
We have revised this section of the discussion, renamed it, and moved it into the Results. It presents a control analysis testing a different assumption about the cost of changing synaptic weight configurations.

3) Similarly, the subtitle "Maximum entropy connectivity distributions" is not clear. What questions are being addressed here?
This section presented an alternate motivation for the degree distribution models that we considered. In the interest of focus and clarity, we have removed it.

4) In Equation 21, the second sum runs from $n_2 = n_1 + 1$, not from $n_2 = n_1$.
Corrected, thank you.

5) In equation 24, the left hand side should not have index i.
Corrected (and the same typo in subsequent equations also corrected).

6) Please break equation 54 in two lines. It falls off the margins.
Done, thank you.

7) The details of derivation of the Laplace approximation is not necessary (although it doesn't hurt either.) More interesting would be asymptotic approximations of all the results for large N and K. This will give an intuition as to how the two models are different.
We have added a couple sentences interpreting the large-K approximations of the modes of the bounded and fixed net weight models in the Results section "Optimally flexible degree distributions".

References

1. Albus JS. A theory of cerebellar function. Mathematical Biosciences. 1971;10(1):25–61. doi:10.1016/0025-5564(71)90051-4.

2. Marr D. A theory of cerebellar cortex. The Journal of Physiology. 1969;202(2):437–470.

3. Hopfield JJ. Neural networks and physical systems with emergent collective computational abilities. Proceedings of the National Academy of Sciences. 1982;79(8):2554–2558.

4. Barak O, Rigotti M, Fusi S. The sparseness of mixed selectivity neurons controls the generalization-discrimination trade-off. The Journal of Neuroscience: The Official Journal of the Society for Neuroscience. 2013;33(9):3844–3856. doi:10.1523/JNEUROSCI.2753-12.2013.

5. Rigotti M, Barak O, Warden MR, Wang XJ, Daw ND, Miller EK, et al. The importance of mixed selectivity in complex cognitive tasks. Nature. 2013;497(7451):585–590. doi:10.1038/nature12160.

6. Babadi B, Sompolinsky H. Sparseness and Expansion in Sensory Representations. Neuron. 2014;83(5):1213–1226. doi:10.1016/j.neuron.2014.07.035.

7. Litwin-Kumar A, Harris KD, Axel R, Sompolinsky H, Abbott LF. Optimal Degrees of Synaptic Connectivity. Neuron. 2017;93(5):1153–1164.e7. doi:10.1016/j.neuron.2017.01.030.

8. Brunel N, Hakim V, Isope P, Nadal JP, Barbour B. Optimal Information Storage and the Distribution of Synaptic Weights: Perceptron versus Purkinje Cell. Neuron. 2004;43(5):745–757. doi:10.1016/j.neuron.2004.08.023.

9. Chapeton J, Fares T, LaSota D, Stepanyants A. Efficient associative memory storage in cortical circuits of inhibitory and excitatory neurons. Proceedings of the National Academy of Sciences. 2012;109(51):E3614–E3622. doi:10.1073/pnas.1211467109.

10. Brunel N. Is cortical connectivity optimized for storing information? Nature Neuroscience. 2016;19(5):749–755. doi:10.1038/nn.4286.

11. Pereira U, Brunel N. Attractor Dynamics in Networks with Learning Rules Inferred from In Vivo Data. Neuron. 2018;99(1):227–238.e4. doi:10.1016/j.neuron.2018.05.038.

12. Zhang D, Zhang C, Stepanyants A. Robust Associative Learning Is Sufficient to Explain the Structural and Dynamical Properties of Local Cortical Circuits. Journal of Neuroscience. 2019;39(35):6888–6904. doi:10.1523/JNEUROSCI.3218-18.2019.

13. Kasai H, Matsuzaki M, Noguchi J, Yasumatsu N, Nakahara H. Structure-stability-function relationships of dendritic spines. Trends in Neurosciences. 2003;26(7):360–368. doi:10.1016/S0166-2236(03)00162-0.

14. Turrigiano GG, Leslie KR, Desai NS, Rutherford LC, Nelson SB. Activity-dependent scaling of quantal amplitude in neocortical neurons. Nature. 1998;361:892–896.

15. Desai NS, Cudmore RH, Nelson SB, Turrigiano GG. Critical periods for experience-dependent synaptic scaling in visual cortex. Nature Neuroscience. 2002;5(8):783–789. doi:10.1038/nn878.

16. Goel A, Jiang B, Xu LW, Song L, Kirkwood A, Lee HK. Cross-modal regulation of synaptic AMPA receptors in primary sensory cortices by visual experience. Nature Neuroscience. 2006;9(8):1001–1003. doi:10.1038/nn1725.

17. Goold CP, Nicoll RA. Single-Cell Optogenetic Excitation Drives Homeostatic Synaptic Depression. Neuron. 2010;68(3):512–528. doi:10.1016/j.neuron.2010.09.020.

18. Vale C, Sanes DH. The effect of bilateral deafness on excitatory and inhibitory synaptic strength in the inferior colliculus. European Journal of Neuroscience. 2002;16(12):2394–2404. doi:10.1046/j.1460-9568.2002.02302.x.

19. Hartman KN, Pal SK, Burrone J, Murthy VN. Activity-dependent regulation of inhibitory synaptic transmission in hippocampal neurons. Nature Neuroscience. 2006;9(5):642–649. doi:10.1038/nn1677.

20. Joseph A, Turrigiano GG. All for One But Not One for All: Excitatory Synaptic Scaling and Intrinsic Excitability Are Coregulated by CaMKIV, Whereas Inhibitory Synaptic Scaling Is Under Independent Control. The Journal of Neuroscience: The Official Journal of the Society for Neuroscience. 2017;37(28):6778–6785. doi:10.1523/JNEUROSCI.0618-17.2017.

21. Maffei A, Nelson SB, Turrigiano GG. Selective reconfiguration of layer 4 visual cortical circuitry by visual deprivation. Nature Neuroscience. 2004;7(12):1353–1359. doi:10.1038/nn1351.

22. Kilman V, Rossum MCWv, Turrigiano GG. Activity Deprivation Reduces Miniature IPSC Amplitude by Decreasing the Number of Postsynaptic GABAA Receptors Clustered at Neocortical Synapses. Journal of Neuroscience. 2002;22(4):1328–1337. doi:10.1523/JNEUROSCI.22-04-01328.2002.

23. Hengen KB, Lambo ME, Van Hooser SD, Katz DB, Turrigiano GG. Firing rate homeostasis in visual cortex of freely behaving rodents. Neuron. 2013;80(2):335–342. doi:10.1016/j.neuron.2013.08.038.

24. Heisenberg M. Mushroom body memoir: from maps to models. Nature Reviews Neuroscience. 2003;4(4):266–275. doi:10.1038/nrn1074.

25. Owald D, Waddell S. Olfactory learning skews mushroom body output pathways to steer behavioral choice in Drosophila. Current Opinion in Neurobiology. 2015;35:178–184. doi:10.1016/j.conb.2015.10.002.

26. Murthy M, Fiete I, Laurent G. Testing odor response stereotypy in the Drosophila mushroom body. Neuron. 2008;59(6):1009–1023. doi:10.1016/j.neuron.2008.07.040.

27. Caron SJC, Ruta V, Abbott LF, Axel R. Random convergence of olfactory inputs in the *Drosophila* mushroom body. Nature. 2013;497(7447):113–117. doi:10.1038/nature12063.

28. Stevens CF. What the fly's nose tells the fly's brain. Proceedings of the National Academy of Sciences. 2015;112(30):9460–9465. doi:10.1073/pnas.1510103112.

29. Srinivasan S, Greenspan RJ, Stevens CF, Grover D. Deep(er) Learning. Journal of Neuroscience. 2018;38(34):7365–7374. doi:10.1523/JNEUROSCI.0153-18.2018.

30. Grueber WB, Jan LY, Jan YN. Tiling of the Drosophila epidermis by multidendritic sensory neurons. Development (Cambridge, England). 2002;129(12):2867–2878.

31. Keshishian H, Chiba A, Chang TN, Halfon MS, Harkins EW, Jarecki J, et al. Cellular mechanisms governing synaptic development in Drosophila melanogaster. Journal of Neurobiology. 1993;24(6):757–787. doi:10.1002/neu.480240606.

32. Gerhard S, Andrade I, Fetter RD, Cardona A, Schneider-Mizell CM. Conserved neural circuit structure across Drosophila larval development revealed by comparative connectomics. eLife. 2017;6:e29089. doi:10.7554/eLife.29089.

33. Tripodi M, Evers JF, Mauss A, Bate M, Landgraf M. Structural Homeostasis: Compensatory Adjustments of Dendritic Arbor Geometry in Response to Variations of Synaptic Input. PLOS Biology. 2008;6(10):e260. doi:10.1371/journal.pbio.0060260.

34. Kremer MC, Christiansen F, Leiss F, Paehler M, Knapek S, Andlauer TFM, et al. Structural Long-Term Changes at Mushroom Body Input Synapses. Current Biology. 2010;20(21):1938–1944. doi:10.1016/j.cub.2010.09.060.

35. Pech U, Revelo NH, Seitz KJ, Rizzoli SO, Fiala A. Optical dissection of experience-dependent pre- and postsynaptic plasticity in the Drosophila brain. Cell Reports. 2015;10(12):2083–2095. doi:10.1016/j.celrep.2015.02.065.

36. Doll CA, Vita DJ, Broadie K. Fragile X Mental Retardation Protein Requirements in Activity-Dependent Critical Period Neural Circuit Refinement. Current biology: CB. 2017;27(15):2318–2330.e3. doi:10.1016/j.cub.2017.06.046.

37. Eichler K, Li F, Litwin-Kumar A, Park Y, Andrade I, Schneider-Mizell CM, et al. The complete connectome of a learning and memory centre in an insect brain. Nature. 2017;548(7666):175–182. doi:10.1038/nature23455.

38. Takemura Sy, Aso Y, Hige T, Wong A, Lu Z, Xu CS, et al. A connectome of a learning and memory center in the adult Drosophila brain. eLife. 2017;6:e26975. doi:10.7554/eLife.26975.

39. Barber D. Bayesian Reasoning and Machine Learning; 2012. Available from: /core/books/bayesian-reasoning-and-machine-learning/37DAFA214EEE41064543384033D2ECF0.

40. Harris KM, Stevens JK. Dendritic spines of CA 1 pyramidal cells in the rat hippocampus: serial electron microscopy with reference to their biophysical characteristics. The Journal of Neuroscience: The Official Journal of the Society for Neuroscience. 1989;9(8):2982–2997.

41. Harris KM, Sultan P. Variation in the number, location and size of synaptic vesicles provides an anatomical basis for the nonuniform probability of release at hippocampal CA1 synapses. Neuropharmacology. 1995;34(11):1387–1395.

42. Schikorski T, Stevens CF. Quantitative Ultrastructural Analysis of Hippocampal Excitatory Synapses. Journal of Neuroscience. 1997;17(15):5858–5867. doi:10.1523/JNEUROSCI.17-15-05858.1997.

43. Schikorski T, Stevens CF. Morphological correlates of functionally defined synaptic vesicle populations. Nature Neuroscience. 2001;4(4):391–395. doi:10.1038/86042.

44. Bourne JN, Chirillo MA, Harris KM. Presynaptic ultrastructural plasticity along CA3→CA1 axons during long-term potentiation in mature hippocampus. The Journal of Comparative Neurology. 2013;521(17):3898–3912. doi:10.1002/cne.23384.

45. Cuntz H, Borst A, Segev I. Optimization principles of dendritic structure. Theoretical Biology and Medical Modelling. 2007;4(1):21. doi:10.1186/1742-4682-4-21.

46. Jaffe DB, Carnevale NT. Passive Normalization of Synaptic Integration Influenced by Dendritic Architecture. Journal of Neurophysiology. 1999;82(6):3268–3285. doi:10.1152/jn.1999.82.6.3268.

47. Chitwood RA, Hubbard A, Jaffe DB. Passive electrotonic properties of rat hippocampal CA3 interneurones. The Journal of Physiology. 1999;515(3):743–756. doi:10.1111/j.1469-7793.1999.743ab.x.

48. Magee JC, Cook EP. Somatic EPSP amplitude is independent of synapse location in hippocampal pyramidal neurons. Nature Neuroscience. 2000;3(9):895–903. doi:10.1038/78800.

49. Chowdhury D, Hell JW. Homeostatic synaptic scaling: molecular regulators of synaptic AMPA-type glutamate receptors. F1000Research. 2018;7. doi:10.12688/f1000research.13561.1.

50. Wen Q, Chklovskii DB. A Cost–Benefit Analysis of Neuronal Morphology. Journal of Neurophysiology. 2008;99(5):2320–2328. doi:10.1152/jn.00280.2007.

51. Zhu S, Chiang AS, Lee T. Development of the Drosophila mushroom bodies: elaboration, remodeling and spatial organization of dendrites in the calyx. Development (Cambridge, England). 2003;130(12):2603–2610. doi:10.1242/dev.00466.

52. Lin HH, Lai JSY, Chin AL, Chen YC, Chiang AS. A Map of Olfactory Representation in the Drosophila Mushroom Body. Cell. 2007;128(6):1205–1217. doi:10.1016/j.cell.2007.03.006.
